# The F130S point mutation in the Arabidopsis high-affinity K^+^ transporter AtHAK5 increases K^+^ over Na^+^ and Cs^+^ selectivity and confers Na^+^ and Cs^+^ tolerance to yeast under heterologous expression

**DOI:** 10.3389/fpls.2014.00430

**Published:** 2014-09-02

**Authors:** Fernando Alemán, Fernando Caballero, Reyes Ródenas, Rosa M. Rivero, Vicente Martínez, Francisco Rubio

**Affiliations:** Centro de Edafología y Biología Aplicada del Segura-CSICMurcia, Spain

**Keywords:** potassium, sodium, cesium, selectivity, HAK, Arabidopsis, random mutagenesis, point mutation

## Abstract

Potassium (K^+^) is an essential macronutrient required for plant growth, development and high yield production of crops. Members of group I of the KT/HAK/KUP family of transporters, such as HAK5, are key components for K^+^ acquisition by plant roots at low external K^+^ concentrations. Certain abiotic stress conditions such as salinity or Cs^+^-polluted soils may jeopardize plant K^+^ nutrition because HAK5-mediated K^+^ transport is inhibited by Na^+^ and Cs^+^. Here, by screening in yeast a randomly-mutated collection of AtHAK5 transporters, a new mutation in AtHAK5 sequence is identified that greatly increases Na^+^ tolerance. The single point mutation F130S, affecting an amino acid residue conserved in HAK5 transporters from several species, confers high salt tolerance, as well as Cs^+^ tolerance. This mutation increases more than 100-fold the affinity of AtHAK5 for K^+^ and reduces the *K*_i_ values for Na^+^ and Cs^+^, suggesting that the F130 residue may contribute to the structure of the pore region involved in K^+^ binding. In addition, this mutation increases the *V*_max_ for K^+^. All this changes occur without increasing the amount of the AtHAK5 protein in yeast and support the idea that this residue is contributing to shape the selectivity filter of the AtHAK5 transporter.

## Introduction

Potassium (K^+^) is an essential nutrient for plants, required for plant growth and development (Marschner, 2012). It fulfills important functions related to enzyme activation, protein synthesis, maintenance of cytoplasmic pH and transmembrane voltage gradients and neutralization of negative charges (Maathuis, 2009). It is the most abundant cationic component, accounting for more than a 10% of the plant dry weight (White and Karley, 2010). As it occurs with other nutrients, roots take up K^+^ from the soil solution through specific transport systems at the plasma membrane of epidermal and cortical cells (Marschner, 2012). Physiological and molecular studies in the model plant *Arabidopsis thaliana* have described two systems, AtHAK5 and AKT1, a high-affinity K^+^ transporter and an inward rectifying K^+^ channel respectively, which are the major contributors to root K^+^ uptake (Alemán et al., 2011; Nieves-Cordones et al., 2014). The relative contribution of these two systems to K^+^ nutrition depends on the external K^+^ concentration and several other factors such as other external ions or root cell membrane potential. At high K^+^ concentrations (around 1 mM), within the low-affinity system described by Epstein (Epstein, 1966), AKT1 is the only system mediating K^+^ uptake. Below 200 μM K^+^, corresponding to the high-affinity range of concentrations, both transport systems can contribute. However, at very low K^+^ concentrations (<10 μM) the only system involved in K^+^ acquisition is AtHAK5 (Rubio et al., 2008, 2010; Pyo et al., 2010). This model may be extended to other plant species although the relative contribution of each of the two systems over the range of external K^+^ concentration may vary among them. For example, in tomato or pepper plants, the AtHAK5 homologs LeHAK5 or CaHAK1 respectively, dominate K^+^ uptake over the AKT1 homologs at low external concentrations (Martínez-Cordero et al., 2004, 2005; Nieves-Cordones et al., 2007).

Operation of the HAK5-type of transporters may be essential for K^+^ nutrition in K^+^ depleted soils. In addition, K^+^ uptake under abiotic stress conditions such as salinity or Cs^+^-polluted soils may also largely depend on HAK5-type transporters (Hampton et al., 2004; Qi et al., 2008; Alemán et al., 2009). Under salinity, uptake and accumulation of Na^+^ in roots cells depolarize their plasma membrane (Volkov and Amtmann, 2006; Chen et al., 2007; Nieves-Cordones et al., 2008) and reduce the driving force for K^+^ uptake. As a result, AKT1 function may be importantly impaired but, K^+^ uptake through the K^+^-H^+^ symporter mechanism proposed for the HAK5-type transporters, may be still possible (Rodríguez-Navarro, 2000). Supporting this idea it has been reported that the Arabidopsis AtHAK5 is required for K^+^ uptake and plant growth at low K^+^ in the presence of salinity, even though *AtHAK5* expression is reduced under high Na^+^ concentrations (Nieves-Cordones et al., 2010). In Cs^+^-polluted soils, K^+^ uptake through AKT1 may be importantly inhibited because Cs^+^ is a potent blocker of AKT1 channels (Bertl et al., 1997; White and Broadley, 2000).

Although HAK5-type transporters may be the major contributors to K^+^ uptake under Na^+^- or Cs^+^-affected soils, they are inhibited by these cations. Both Na^+^ and Cs^+^ competitively inhibit K^+^ uptake through HAK5-type transporters (Santa-María et al., 1997; Rubio et al., 2000). This leads to two important effects. On one hand, K^+^ supply is reduced and in fact, it has been described that high external Na^+^ or Cs^+^ may lead to K^+^ deficiency (Botella et al., 1997; Carden et al., 2003; Hampton et al., 2004; Shabala et al., 2006; Adams et al., 2013). On the other hand, Na^+^ and Cs^+^ may accumulate into the cell by entering through HAK5 transporters (Santa-María et al., 1997; Rubio et al., 2000; Qi et al., 2008). Overall, pivotal parameters related to Na^+^ or Cs^+^ tolerance such as the cytoplasmic K^+^/Na^+^ (Maathuis and Amtmann, 1999; Shabala and Cuin, 2008) or K^+^/Cs^+^ (Hampton et al., 2004; Adams et al., 2013) ratios are reduced and plant growth is inhibited.

These three stress conditions, K^+^-depleted, salt-affected or Cs^+^-polluted soils, that may make K^+^ nutrition largely dependent on HAK5 operation, affect agriculture worldwide. Many agricultural lands are intrinsically deficient in K^+^ (Rengel and Damon, 2008; Römheld and Kirkby, 2010) or become K^+^ deficient because of an inadequate fertilization that prioritize N and P over K fertilization (Römheld and Kirkby, 2010). Salinity affects more than 20% of irrigated lands worldwide and, because most crop species are glycophytes, it reduces crop productivity (Munns and Tester, 2008). Finally, although the natural concentration of Cs^+^ in soils is low, certain areas may contain Cs^+^ concentrations that reduce plant growth because Cs^+^ is toxic to plants. In addition, agricultural lands affected by discharges of nuclear power plants result in polluted soils with radiocesium, which readily enters the food chain (White and Broadley, 2000).

Obtaining plants with more selective HAK5 transporters may be a solution to these agricultural problems (Niu et al., 1995). This requires function-structure relationship studies, which are scarce in literature. Some studies reported the isolation of mutated plant transporters of the HAK5-type that when expressed in yeast showed altered kinetics of K^+^ uptake (Rubio et al., 2000; Senn et al., 2001; Garciadeblas et al., 2007; Mangano et al., 2008; Nieves-Cordones et al., 2008). Here we have focused on the identification of amino acid residues that are important for K^+^ over Na^+^ or Cs^+^ selectivity of the Arabidopsis AtHAK5 transporter. Mutagenic PCR followed by selection in yeast allowed us for the identification of a highly conserved amino acid residue in the family of HAK transporters, F130, that is probably involved in the selectivity of the transporter for K^+^.

## Materials and methods

### Yeast and bacterial strains and growth media

*Saccharomyces cerevisiae* yeast strain 9.3 (*MAT***a**, *ena1D:HIS3::ena4D, leu2, ura3–1, trp1–1, ade2–1, trk1D, trk2::pCK64*) (Bañuelos et al., 1995) with deleted TRK1 and TRK2 K^+^ uptake systems and ENA1-ENA4 Na^+^ extrusion Na^+^-ATPases was used throughout the study. The *Escherichia coli* strain DH5α (Hanahan, 1985) was used for plasmid DNA amplification. For yeast growth YPD (1% yeast extract, 2% peptone, 2% glucose), SD (Sherman, 1991) and Arginine Phosphate medium (AP) (Rodríguez-Navarro and Ramos, 1984) supplemented with KCl, NaCl and requirements as indicated were used. For bacterial growth, LB media supplemented with ampicillin as required was used. Yeast transformation was carried out by the high-efficiency protocol of the lithium acetate/single-stranded carrier DNA/PEG method of transformation of *S. cerevisiae* (Gietz and Schiestl, 2007). Standard procedures were used for *E. coli* growth and transformation, and DNA manipulations (Sambrook and Russell, 2001).

For determining doubling times of yeast cells, liquid cultures were used. The optical density at 550 nm of the yeast cultures was determined in a spectrophotometer at different time points during 72 h. The optical density vs. time was plotted and the doubling time (the time required to duplicate the optical density) calculated during the exponential growth phase.

### Random AtHAK5 mutagenesis, mutant selection and site-directed mutagenesis

The AtHAK5 cDNA was mutagenized randomly by error prone PCR (epPCR) as described by Wong et al. (2007) with minor modifications. In short, two epPCRs biased toward transitions or transversions were carried out to amplify AtHAK5 cDNA to get a broader range of possible amino acid substitutions (Table S1). The primers used to amplify the AtHAK5 cDNA were Forward: 5′-AAAAAATATACCCCAGCCTCG-3′ and Reverse 5′-GGCGAAGAAGTCCAAAGCTGG-3, which can bind up- and downstream regions respectively of the NotI and BamHI pDR195 cloning sites (Rentsch et al., 1995), where the cDNA of AtHAK5 was inserted.

The amplified cDNA fragments and a NotI-BamHI digested pDR195 plasmid were cotransformed into yeast to allow homologous recombination (ratio 10:1 epPCR:vector) as previously described (Rubio et al., 1999). The amplified cDNA and the plasmid shared a region of more than 50 base pairs on each end to allow proper homologous recombination (Muhlrad et al., 1992). Transformants were first selected on SD medium lacking uracil and supplemented with 100 mM KCl and then replica-plated to AP medium supplemented with 0.1 mM KCl plus different concentrations of Na^+^. The best growing colonies were selected, and their plasmids were isolated and reintroduced into yeast. Yeast retransformants that confirmed growth on high Na^+^ were selected, their plasmids isolated and the AtHAK5 cDNA sequenced. To obtain single point mutations, PCR-based site directed mutagenesis was followed as described elsewhere (Cadwell and Joyce, 1992).

### Internal ionic content determination in yeast

Internal K^+^, Na^+^, and Cs^+^ concentrations in yeast were determined after 24 h of growth in liquid AP media supplemented with K^+^, Na^+^, and Cs^+^ as indicated. In short, samples of yeast cultures were filtered through a 0.8-μm-pore nitrocellulose membrane filter (Millipore, Bedford, MA) and washed with 20 mM MgCl_2_. Ionic contents were extracted after 24 h incubation in 0.1 M HCl. The K^+^, Na^+^, or Cs^+^ concentrations were determined by atomic emission spectrophotometry of the acid extracts from the cells (Rodríguez-Navarro and Ramos, 1984).

### Rb^+^ uptake experiments

For kinetic characterization of K^+^ uptake, Rb^+^ was used in uptake experiments (Rodríguez-Navarro, 2000). Yeast cells of the 9.3 strain transformed with plasmids harboring WT AtHAK5 and the different mutants were grown overnight in AP liquid media supplemented with 3 mM K^+^ at 28°C with shaking. After overnight incubation, cell cultures reached an optical density (550) of 0.2–0.3. Then, cells were collected by centrifugation and suspended in fresh AP media without K^+^, and incubated for 6 h. After this 6 h period of K^+^ starvation cells were collected by centrifugation and suspended in uptake buffer that contained 10 mM MES, 0.1 mM MgCl_2_ and 2% glucose brought to pH 6,0 with Ca(OH)_2_. Cells were placed at 28°C in a shaker bath and at time zero Rb^+^ was added. Samples were taken at different time points during 10 min and collected on nitrocellulose 0.8 μm pore filters that were incubated overnight in 5 mL of 0.1 M HCl to extract their internal Rb^+^, which was determined by atomic emission spectrophotometry. The internal Rb^+^ concentrations per unit of cell dry weight were plotted against time and the initial rates of Rb^+^ uptake were calculated on a dry weight basis and per unit of time (Rodríguez-Navarro and Ramos, 1984). The rates of Rb^+^ uptake were plotted at the different external concentrations of Rb^+^ employed and fitted to Michaelis-Menten equations to calculate the kinetic parameters. These experiments were carried out with cultures from several independent transformants producing the same results.

### K^+^ and Cs^+^ depletion experiments

For determining the *V*_max_ of K^+^ and Cs^+^ uptake, depletion experiments were performed. Yeast cells were grown and starved of K^+^ as described for the Rb^+^ uptake experiments and then transferred to AP media to reach an optical density of 2. At zero time, K^+^ or Cs^+^ was added to reach a 50 μM concentration. Samples of the cell suspensions were taken at different time points and centrifuged to spin down cells. K^+^ or Cs^+^ concentrations were determined in the supernatant by atomic emission spectrophotometry. The K^+^ or Cs^+^ uptake rates were calculated from the difference in the external cation concentration between two time points per unit of dry weight and unit of time.

### Protein extraction and western blot

Yeast cells expressing the different AtHAK5 versions as well as the empty plasmid were grown overnight in YPD media supplemented with 100 mM K^+^. Total membranes from yeast were isolated as described elsewhere (Serrano, 1988). 25 μg of proteins from total membranes of each yeast strain were subjected to SDS/PAGE and immunoblotting following standard procedures (Sambrook and Russell, 2001). AtHAK5 protein of a predicted mass of 87.85 kDa was immunodetected with a polyclonal antibody raised in rabbit against the YGYKEDIEEPDEFE peptide present in AtHAK5 amino acid sequence, synthesized by GenScript (Piscataway, NJ USA). After immunological detection, the PVDF membrane was washed and stained with comassie blue.

## Results

### Selection of AtHAK5 mutants that increase tolerance to NaCl

To isolate novel HAK5 mutants, the Na^+^ sensitive yeast strain 9.3, lacking functional TRK1 and TKR2 K^+^ uptake systems as well as ENA1-ENA4 Na^+^ extruding ATPases, was used for heterologous expression (Bañuelos et al., 1995). Plasmids containing putative mutants of AtHAK5 were obtained by allowing homologous recombination in yeast of mutated cDNA and plasmid fragments that rescued the ura^−^ mutation of the 9.3 yeast strain, as described in Materials and Methods Section. Colonies growing in SD-URA selective media supplemented with 100 mM K^+^ were replica plated to different selective minimal AP media. These selective media contained 0.1 mM K^+^ with no added Na^+^ or with 400 mM Na^+^. Thirty five colonies that showed a better growth in the presence of Na^+^ were selected for further analysis. To ensure that the best performance of the selected yeast colonies was promoted by the plasmid and not by a reversion of the yeast genetic background, the plasmids were recovered from yeast cells and retransformed into the 9.3 yeast strain. The capacity of the retransformants to grow in the presence of high Na^+^ was checked. The 9.3 strain transformed with the WT AtHAK5 was used as a control. However, as previously shown (Rubio et al., 2000), the low *V*_max_ of K^+^ uptake mediated by WT AtHAK5 in yeast prevented its functional complementation of *trk1, trk2* yeast mutants. Therefore, an additional control that consisted of yeast cells expressing the previously characterized AtHAK5mutant with the L776H mutation was used. This mutation increased the *V*_max_ of K^+^ uptake and allowed growth of *trk1, trk2* yeasts at low K^+^ (Rubio et al., 2000). None of the yeast cells harboring the putative AtHAK5 mutants grew better than those expressing the L776H mutant (not shown).

**Figure 1:**
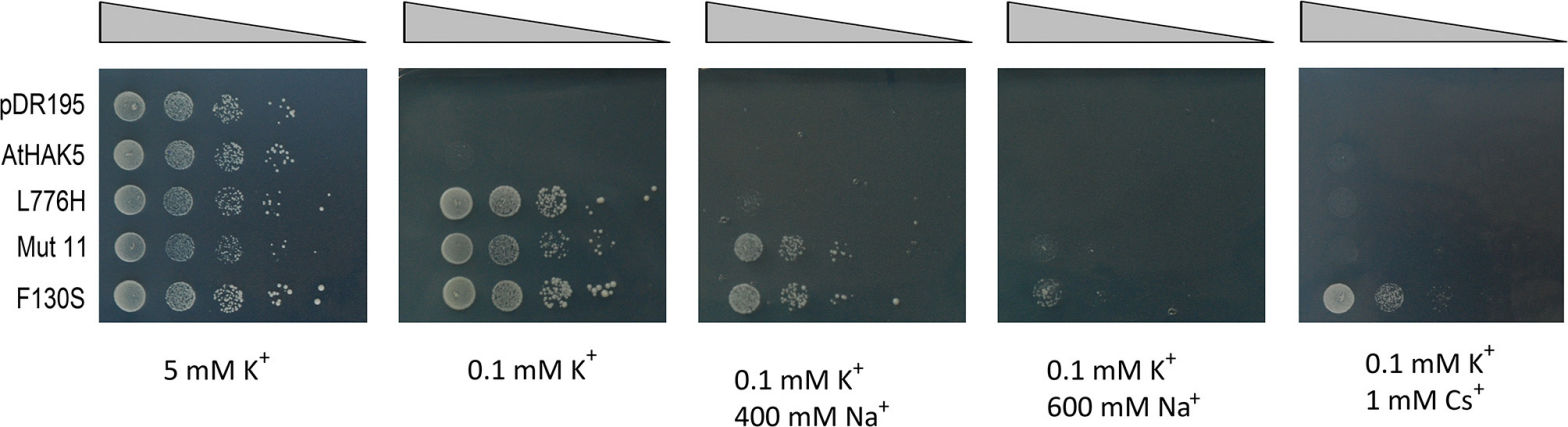
**Growth of 9.3 yeast cells expressing WT and mutated AtHAK5 K^+^ transporters**. Cells of the yeast strain 9.3 (*trk1, trk2, ena1-4*) expressing the empty plasmid pDR195, WT AtHAK5 and the mutants L776H, Mut11 (L776H, F130S) and F130S were used for growth assays on drop tests on solid media. Serial dilutions of cell suspensions were plated on minimal AP media supplemented with the indicated K^+^, Na^+^ and Cs^+^ concentrations.

In order to obtain AtHAK5 mutations that enhanced Na^+^ tolerance beyond that promoted by the L776H mutant, a similar approach was undertaken, but using the cDNA of the L776H mutant as a starting material. The selective pressure was also increased by using higher Na^+^ concentrations in the selective media. In this case, 17 Na^+^-tolerant colonies were selected on media containing 0.1 mM K^+^ and 600 mM Na^+^. After retransformation and selection for growth, one colony, that was named Mut11, was selected because it showed a better growth than the L776H mutant on plates with high Na^+^. The plasmid containing the Mut11 mutant was isolated and the AtHAK5 cDNA sequenced. It was observed that in addition to the L776H mutation, the Mut11 mutant contained the F130S mutation. To study individual effects of each of these mutations, a cDNA that encoded the single mutant F130S was generated by site directed mutagenic PCR, cloned into the pDR195 plasmid and transformed into the 9.3 strain. Growth of yeast cells expressing double and single mutants as well as WT AtHAK5 and the empty plasmid was studied by drop tests on minimal media (Figure 1).

In the presence of 5 mM K^+^, all yeast strains grew well. When the K^+^ concentration was reduced to 0.1 mM K^+^, yeast cells transformed with the empty plasmid (pDR195) or with WT AtHAK5 were unable to grow. Yeast cells expressing any of the mutated versions of AtHAK5 could grow. When 400 mM Na^+^ was added to the 0.1 mM K^+^ medium, only the double Mut11 mutant and the single F130S mutant were able to grow. Increasing Na^+^ to 600 mM completely arrested growth of Mut11. However, at this high Na^+^ concentration, the cells expressing the single mutant F130S were able to grow, although at low rates.

Tolerance to Cs^+^ was also studied. The presence of 1 mM Cs^+^ in the 0.1 mM K^+^ medium completely inhibited growth of all yeast strains with the exception of those expressing the F130S mutation, that showed an outstanding growth capacity.

For a detailed study of growth rates and tolerance to Na^+^ and Cs^+^, liquid cultures were used. The doubling times of the different yeast strains growing in the presence of different levels of K^+^, Na^+^, and Cs^+^ were determined (Table 1). At high K^+^ (3 mM K^+^) all strains grew although mutants did so faster than WT AtHAK5. At low K^+^ (0.1 mM K^+^) only the mutants grew with no significant differences among them. Including high Na^+^ concentrations to the media gave rise to differences in growth rates among the different mutants. In the presence of 100 mM Na^+^ new mutants, Mut11 and F130S, grew better than the L776H mutant. Further increase of Na^+^ to 300 mM inhibited growth of L776H and reduced that of the new mutants. Importantly, the F130S showed a significant higher rate of growth than the double Mut11 mutant. When 1 mM Cs^+^ was present in the growth media, a dramatic reduction of the growth rates was observed for all mutants, with the exception of the F130S that retained a higher rate of growth.

**Table 1:** **Doubling time (h) of 9.3 yeast strain expressing the indicated version of AtHAK5 under different external concentrations of K^+^, Na^+^, and Cs^+^**.

**Treatment**					
**Mutation**	**3 mM K^+^**	**0.1 mM K^+^**	**0.1 mM K^+^ 100 mM Na^+^**	**0.1 mM K^+^ 300 mM Na^+^**	**0.1 mM K^+^ 1 mM Cs^+^**
WT	6.6 ± 0.2^b^	–	–	–	–
L776H	3.6 ± 0.2^a^	4.3 ± 0.2^a^	9.5 ± 0.6^b^	–	30.3 ± 0.9^b^
Mut11	3.9 ± 0.3^a^	3.7 ± 0.2^a^	5.5 ± 0.2^a^	12.4 ± 2^b^	32.7 ± 1.5^b^
F130S	3.4 ± 0.1^a^	3.5 ± 0.1^a^	5.9 ± 0.4^a^	7.3 ± 1.8^a^	6.8 ± 0.4^a^

*Shown are mean values of at least three replicates ± standard error. Different letters indicate significant differences within each column according to Tukey test (p < 0.01)*.

### Internal K^+^ and Na^+^ concentrations of yeast expressing the AtHAK5 mutants under salt stress

**Figure 2:**
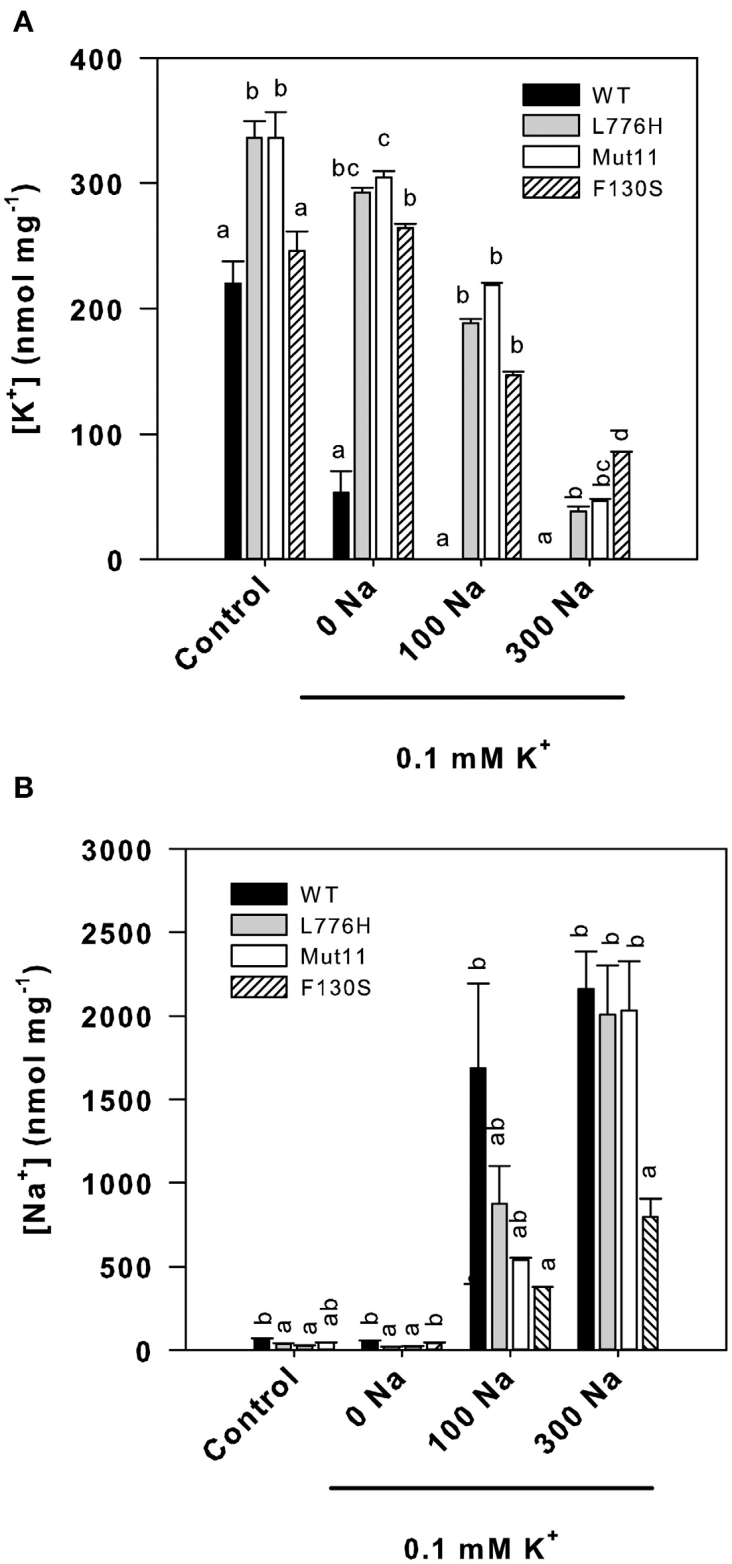
**Internal K^+^ and Na^+^ concentrations of 9.3 yeast cells expressing WT AtHAK5 and mutated AtHAK5 K^+^ transporters**. The same strains described in Figure 1 were grown for 24 h in minimal AP liquid media supplemented with the indicated K^+^ and Na^+^ concentrations. The control medium contained 3 mM K^+^. After 24 h cells were collected by filtration and their ionic content was acid extracted. The K^+^ and Na^+^ concentrations in the acid extracts were determined by atomic emission spectrometry and the internal K^+^ and Na^+^ concentrations calculated on a dry weight basis. Shown are averages of internal K^+^ **(A)** and Na^+^ **(B)** concentrations of at least 6 replicates and error bars denote standard error. Columns with different letters within each ionic treatment are significantly different according to Tukey test (*p* < 0.05).

Internal concentrations of K^+^ and Na^+^ after 24 h of yeast growth in media with different external concentrations of K^+^ and Na^+^ were determined (Figure 2). Under control conditions (3 mM K^+^), all strains exhibited internal K^+^ concentrations above 200 nmol mg^−1^ (Figure 2A). At low external K^+^ (0.1 mM K^+^), the K^+^ concentration of yeast expressing WT AtHAK5 dropped to 52.9 ± 17.4 nmol mg^−1^, whereas yeast expressing the mutants retained a high internal K^+^ (292.4 ± 3.7, 304.8 ± 4.8, and 264.2 ± 3.4 nmol mg^−1^ for L776H, Mut11, and F130S respectively). The presence of 100 mM Na^+^ reduced the internal K^+^ concentrations for WT AtHAK5 to levels below 1 nmol mg^−1^, and no differences among the different mutants were found in their K^+^ concentrations (188.0.4 ± 3.3, 218.6 ± 1.5, and 146.9 ± 2.6 nmol mg^−1^ for L776H, Mut11, and F130S respectively). Further increase of Na^+^ to 300 mM gave rise to differential results. Cells expressing the F130S mutation showed the highest K^+^ concentration (85.6 ± 0.2 nmol mg^−1^) followed by Mut11 (46.4 ± 1.8 nmol mg^−1^) and L776H (38.1 ± 4.0 nmol mg^−1^).

Internal Na^+^ concentrations were also determined (Figure 2B). In the absence of added Na^+^, internal Na^+^ concentrations were very low. Addition of 100 mM Na^+^ increased internal Na^+^ in all cases, resulting the Na^+^ concentrations of cells expressing WT AtHAK5 significantly higher than those expressing the F130S mutant. The presence of 300 mM Na^+^ produced cells with similar high internal Na^+^ concentrations in cells expressing WT AtHAK5, L776H, and Mut11. Importantly, cells expressing the F130S mutation showed a significant lower Na^+^ concentration than the other strains.

**Figure 3:**
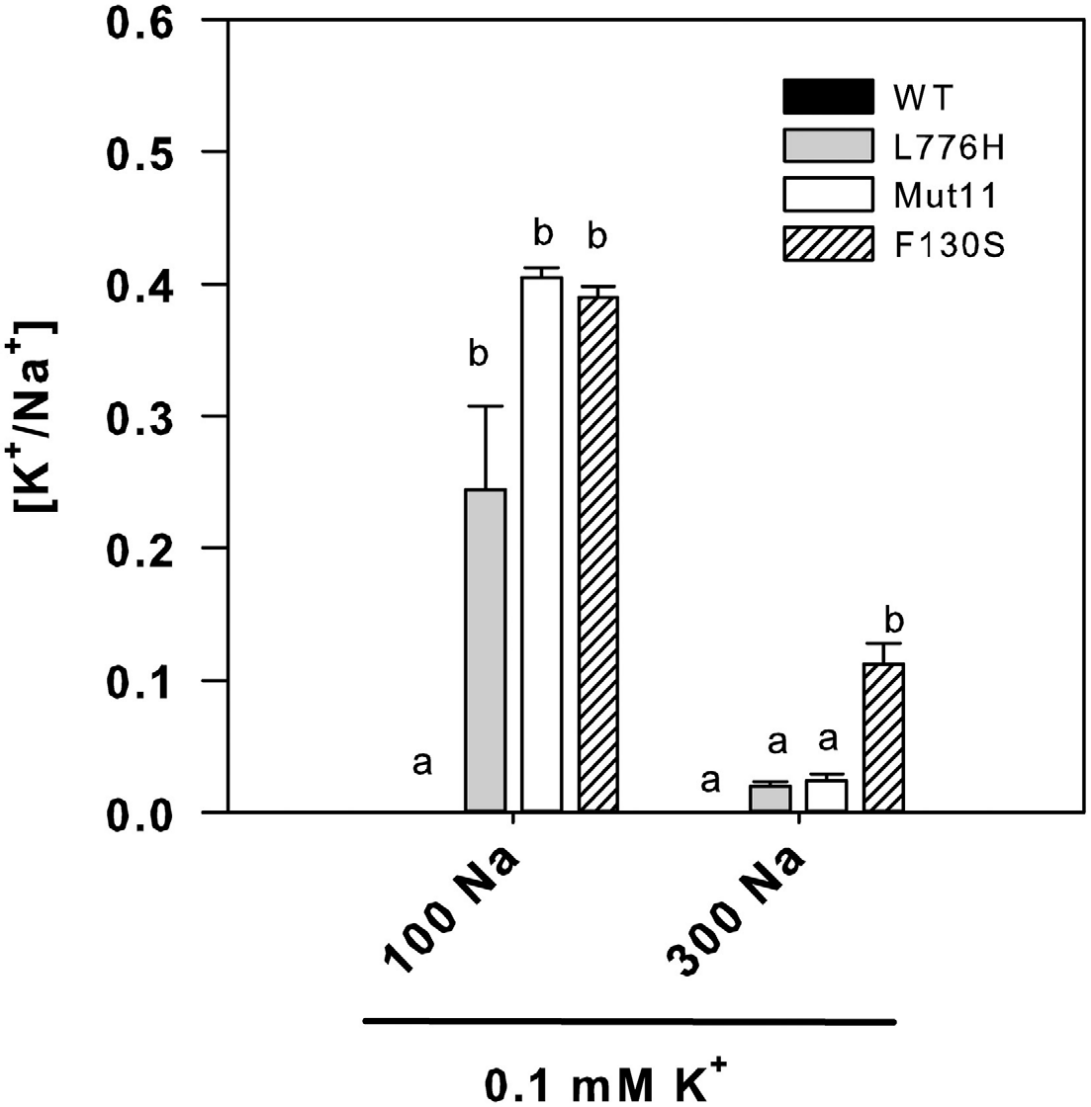
**Internal K^+^/Na^+^ ratios of 9.3 yeast cells expressing WT AtHAK5 and mutated AtHAK5 K^+^ transporters**. Cells were treated and analyzed as indicated in Figure 2. Shown are the ratios of internal K^+^/Na^+^ concentrations of the cells grown in the presence of 0.1 mM K^+^ at 100 or 300 mM Na^+^. Reported values are the average of at least 6 replicates and error bars denote standard error. Columns with different letters within each ionic treatment are significantly different according to Tukey test (*p* < 0.05).

The internal K^+^/Na^+^ ratios were calculated because this is one of the most important parameters for salt tolerance in yeast as well as in plants (Gaxiola et al., 1992; Maathuis and Amtmann, 1999; Gisbert et al., 2000). In the presence of 100 mM Na^+^ (0.1 mM K^+^), cells expressing the AtHAK5 mutants showed similarly higher K^+^/Na^+^ ratios than those expressing WT AtHAK5 (Figure 3). The presence of 300 mM Na^+^ importantly reduced the K^+^/Na^+^ ratio in all cases. WT AtHAK5 and the L776H and Mut11 mutants showed similar low K^+^/Na^+^ ratios. The F130S mutant showed a significantly higher K^+^/Na^+^ ratio than the rest of the yeast strains.

### Internal K^+^ and Cs^+^ concentrations of yeast expressing the mutants under Cs^+^ stress

**Figure 4:**
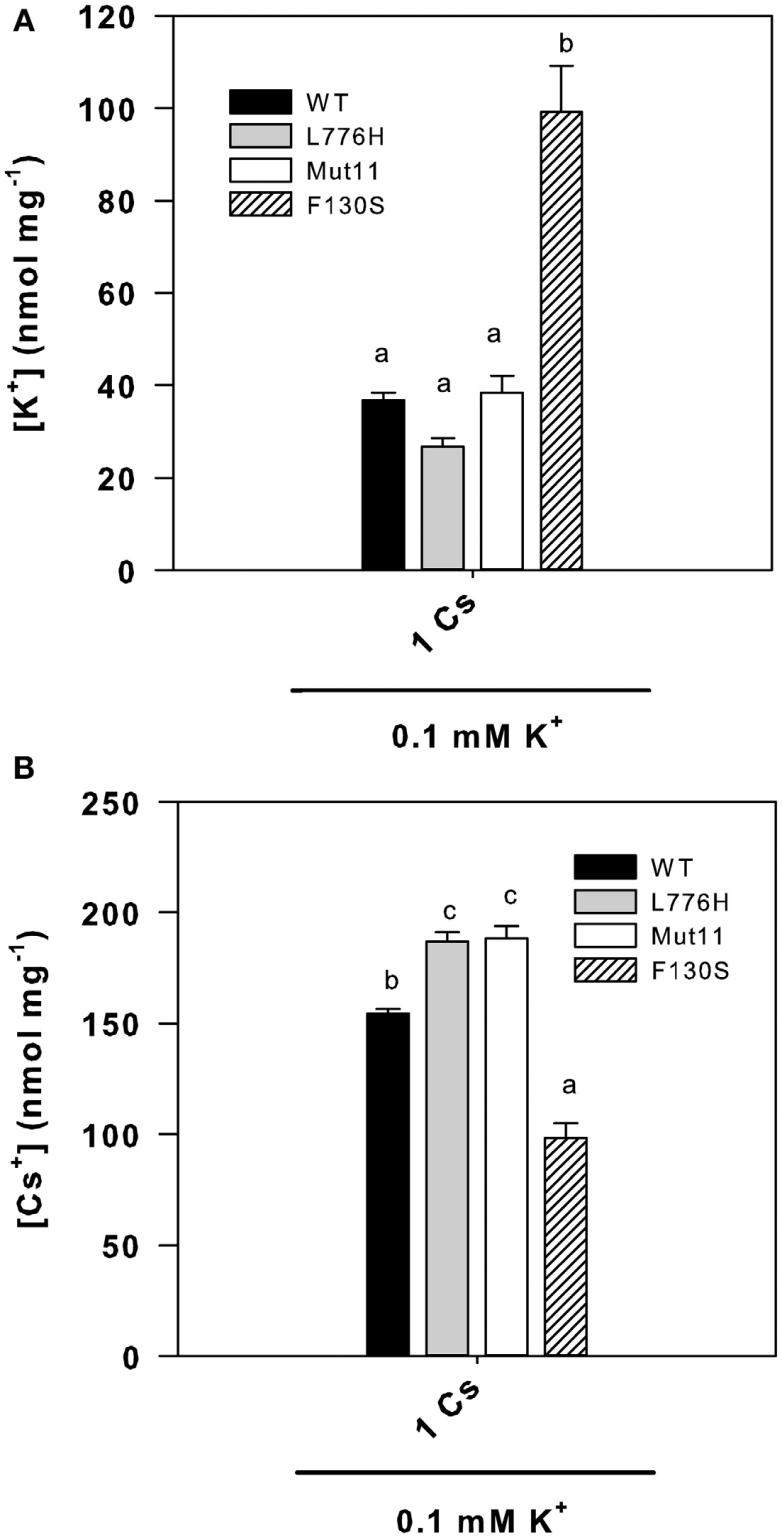
**Internal K^+^ and Cs^+^ concentrations of 9.3 yeast cells expressing WT AtHAK5 and mutated AtHAK5 K^+^ transporters**. The same strains described in Figure 1 were grown for 24 h in minimal AP liquid media supplemented with 0.1 mM K^+^ and 1 mM Cs^+^. After 24 h cells were collected by filtration and their ionic content was acid extracted. The K^+^ and Cs^+^ concentrations in the acid extracts were determined by atomic emission spectrometry and the internal K^+^ and Cs^+^ concentrations calculated on a dry weight basis. Shown are averages of internal K^+^ **(A)** and Cs^+^ **(B)** concentrations of at least 6 replicates and error bars denote standard error. Columns with different letters within each ionic treatment are significant different according to Tukey test (*p* < 0.01).

**Figure 5:**
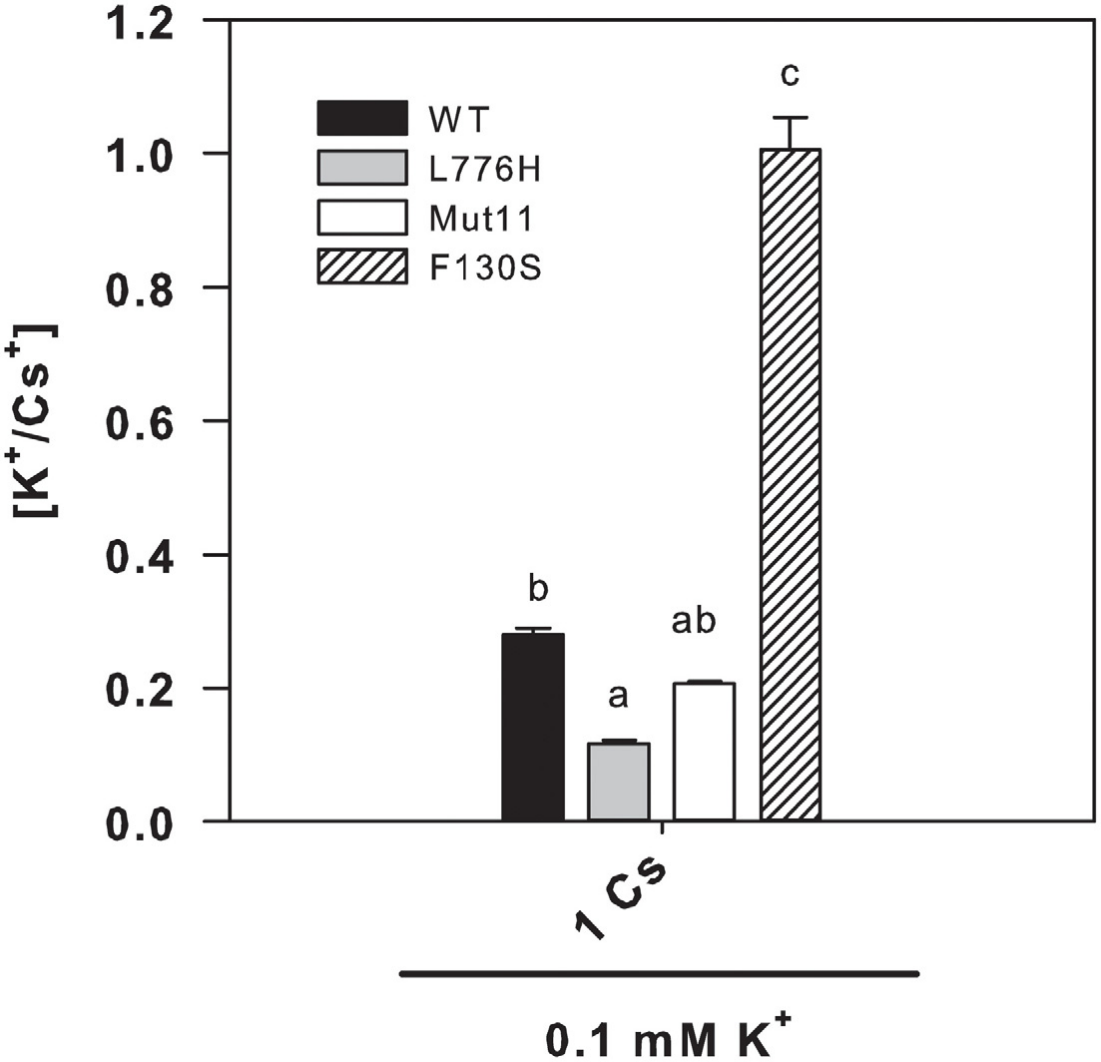
**Internal K^+^/Cs^+^ ratios of 9.3 yeast cells expressing WT AtHAK5 and mutated AtHAK5 K^+^ transporters**. Cells were treated and analyzed as indicated in Figure 4. Shown are the ratios of internal K^+^/Cs^+^ concentrations of the cells grown in the presence of 0.1 mM K^+^ and 1 mM Cs^+^. Reported values are the average of at least 6 replicates and error bars denote standard error. Columns with different letters within each ionic treatment are significantly different according to Tukey test (*p* < 0.01).

Since the F130S mutant showed a remarkable enhanced tolerance to Cs^+^ (Figure 1 and Table 1), we further examined the internal K^+^ and Cs^+^ concentrations in yeast grown for 24 h in liquid medium in the presence of at low K^+^ (0.1 mM K^+^) with 1 mM CsCl. The presence of 1 mM Cs^+^ importantly reduced the internal K^+^ concentrations to 36.6 ± 1.7, 26.7 ± 1.8, 38.4 ± 3.6, and 99.3 ± 9.8 nmol mg^−1^ for WT AtHAK5, L776H, Mut11, and F130S respectively (Figure 4A), far below the internal K^+^ concentrations without Cs^+^ (Figure 2A). However, cells expressing the F130S mutant retained a significantly higher K^+^ concentration than the other yeast strains. When internal Cs^+^ was determined, it was observed that cells expressing the F130S mutant accumulated a significantly lower Cs^+^ concentration than the other strains (Figure 4B). As a result, the F130S mutation led to cells with significantly higher K^+^/Cs^+^ ratios (Figure 5), an important parameter for Cs^+^ tolerance (Hampton et al., 2004).

### Kinetic characterization of AtHAK5 mutants

**Figure 6:**
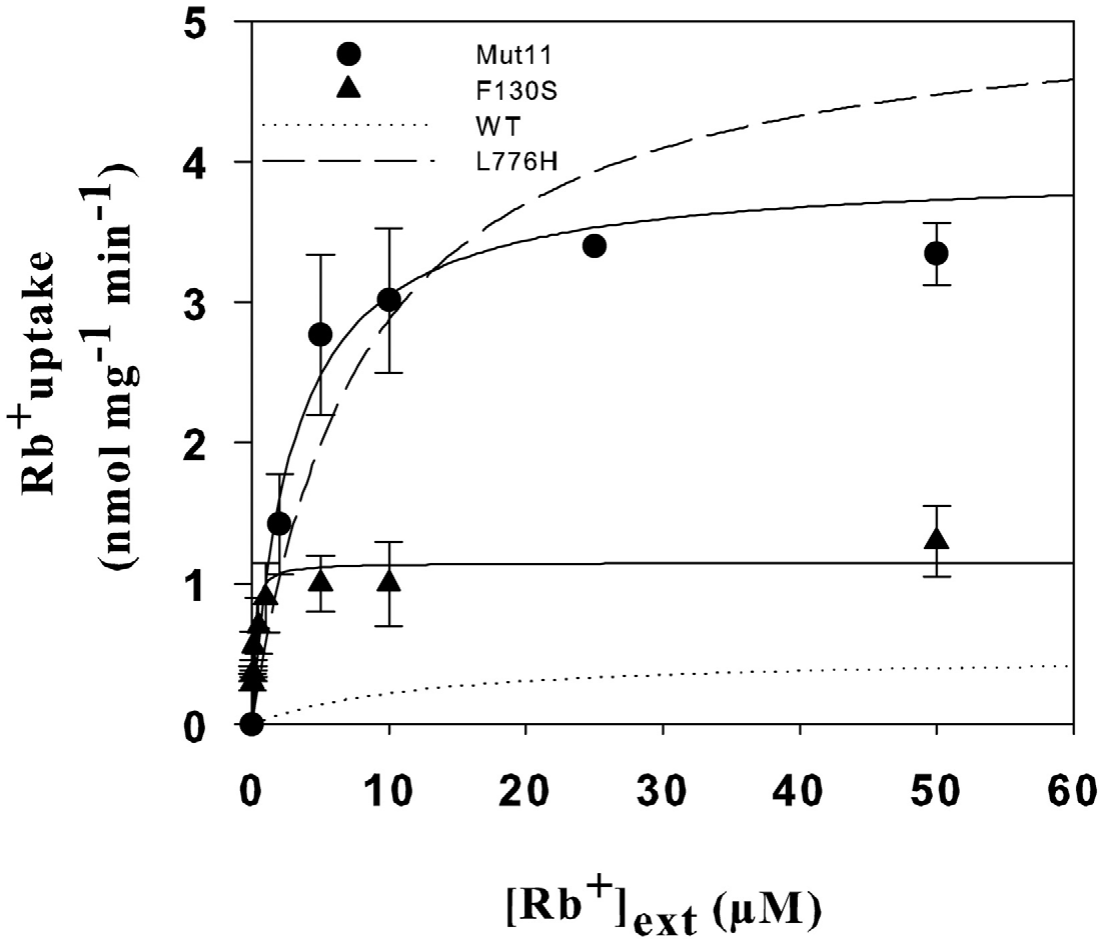
**Initial rates of Rb^+^ uptake as a function of external Rb^+^ in 9.3 cells expressing the AtHAK5 mutants**. Cells of the 9.3 strain expressing the Mut11 and F130S mutants were grown overnight on minimal AP media supplemented with 30 mM K^+^. After 6 h of incubation in K^+^-free AP media cells were transferred to uptake buffer supplemented with different Rb^+^ concentrations. The initial rates of Rb^+^ uptake for each external Rb^+^ concentration were determined and plotted for Mut11 (closed circles) and F130S (closed triangles). Values were fitted to Michaelis-Menten equations. The predicted curves for WT (dotted line) and the L776H mutation (dashed line) are also shown (Rubio et al., 2000).

To gain further insights into the effects that the Mut11 and F130S mutations produced on AtHAK5 functionality, a kinetic characterization was performed. Yeast cells expressing the mutants were grown overnight on AP media supplemented with 3 mM K^+^ and starved of K^+^ for 6 h. Then, Rb^+^ uptake experiments were performed as described in the Materials and Methods Section to calculate the initial rates of Rb^+^ uptake at different external concentrations. The rates of Rb^+^ uptake were plotted against the external Rb^+^ concentration (Figure 6). Clear differences in the uptake kinetics could be observed among the mutants and WT AtHAK5. The data were fitted to Michaelis-Menten equations and *V*_max_ and *K*_m_ values determined (Table 2). All mutants increased the *V*_max_ for Rb^+^ with respect to WT AtHAK5. However, whereas mutants containing the L776H mutation (L776H and Mut11) showed high *V*_max_ values around 10-fold the *V*_max_ of WT AtHAK5, the F130S only showed a 2-fold increase in this parameter. Mut11 showed a reduction of at least 3-fold in the *K*_m_ value with respect to WT AtHAK5. Importantly, the single F130S mutation led to a 100-fold increase in the affinity for Rb^+^ in comparison to WT AtHAK5. The inhibition of Rb^+^ uptake by K^+^, Na^+^ and Cs^+^ was also determined and the *K*_i_ values calculated (Table 2). As with the Rb^+^ *K*_m_ values, the *K*_i_ constants were importantly reduced by the single F130S mutation. In addition, the rates of K^+^ and Cs^+^ depletion from a 50 μM solution were determined. It was observed that cells expressing the F130S depleted K^+^ at higher rates than WT AtHAK5 cells (4.2 ± 0.5 vs 0.6 ± 0.3 nmol mg^−1^ min^−1^) and they depleted Cs^+^ at similar rates compared to WT AtHAK5 cells (2.6 ± 0.7 nmol mg^−1^ min^−1^).

**Table 2:** **Kinetic parameters of Rb^+^ uptake mediated by AtHAK5 and its mutant forms in yeast**.

	***V*max (nmol mg-1 min-1)**	**Rb^+^ Km (μM)**	**K^+^ Ki (μM)**	**Na^+^ Ki(mM)**	**Cs^+^ Ki (μM)**
WT^*^	0.5 ± 0.3	12.6 ± 0.3	12	10	16
L776H^*^	5.2 ± 0.3	8.1 ± 1.0	12	10	16
Mut11	4.1 ± 0.2	2.5 ± 0.7	5.7 ± 0.1	12.3 ± 2.1	21.3 ± 0.8
F130S	1.0 ± 0.2	0.1 ± 0.1	0.3 ± 0.2	0.6 ± 0.3	0.3 ± 0.2

* *From (Rubio et al., 2000)*.

*Values at the average of at least three replicates ± standard error. *V*_max_ (nmol mg^−1^ min^−1^) and *K*_m_ (μM) values for Rb^+^ uptake and *K*_i_ values for inhibition of Rb^+^ uptake by K^+^ (μM), Na^+^ (mM), and and Cs^+^ (μM)*.

### Detection of AtHAK5 and AtHAK5 mutant proteins in yeast

**Figure 7:**
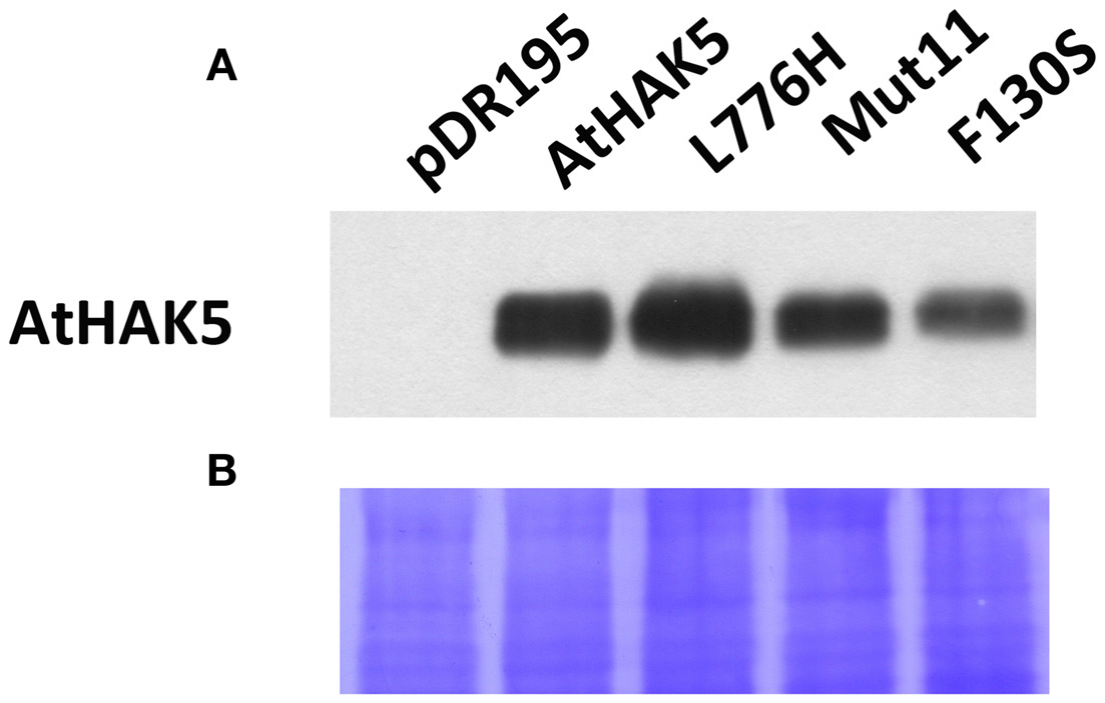
**Detection of the AtHAK5 WT and mutated proteins expressed in yeast by Western blot**. The yeast strains described in Figure 1 were grown overnight in YPD media supplemented with 100 mM K^+^. Then, proteins of the total membrane fraction were isolated, resolved on polyacrylamide gel and transferred to a PVDF membrane for AtHAK5 detection by Western-bolt. A polyclonal antibody raised in rabbit against the YGYKEDIEEPDEFE peptide of AtHAK5 was used. **(A)** Shows the immunological detection of AtHAK5 in the different yeast strains. **(B)** Shows the coomasie blue staining of the hybridized PDVF membrane.

The AtHAK5 protein of the different mutant versions as well as of WT AtHAK5 were immunologically detected. Proteins from total yeast membranes were extracted from liquid yeast cultures and resolved by SDS-PAGE. The AtHAK5 protein was detected by Western blot with a polyclonal antibody raised against AtHAK5. Yeast cells transformed with the empty plasmids did not produce any detectable signal (Figure 7A) whereas those expressing WT AtHAK5 and AtHAK5 mutants produced a signal band corresponding to the AtHAK5 size (predicted mass of 87.85 kDa). Differences in the amount of AtHAK5 with respect to WT AtHAK5 could be detected among the different yeast strains. L776H increased the amount of AtHAK5 protein, Mut11 showed a similar protein accumulation whereas the single F130S mutant showed a reduced amount of AtHAK5. Coomassie staining of the proteins transferred to the hybridized PVDF membrane showed that all lines contained the same amount of total protein (Figure 7B).

## Discussion

In plants, four families of K^+^ transport systems have been described: Shaker channels, KCO channels, HKT transporters and HAK transporters (Very and Sentenac, 2003). The members of the first three families bear putative pore forming regions derived from a K^+^ channel ancestor subunit, region that is present in all K^+^ channels among kingdoms (Mackinnon, 2003; Very and Sentenac, 2003; Corratgé-Faillie et al., 2010; Benito et al., 2014). This explains the high number of structure-function relationship studies on this type of transporters and the deep knowledge of their functional domains. However, this conserved pore forming region is absent in HAK transporters that, together with the lack of HAK representatives in the animal kingdom, place this family of transporters in a distinct group. This, and the scarce structure-function relationship studies on HAK transporters, make the identification of their functional domains an interesting and almost unexplored field of research. We have here undertaken the identification of mutants in the Arabidopsis AtHAK5 that help the identification of those unknown functional domains.

A previous study showed that introducing into the barley high-affinity K^+^ transporter HvHAK1 (Santa-María et al., 1997) amino acids of the N-terminus of the barley low-affinity K^+^ transporter HvHAK2, reduced the affinity of HvHAK1 for K^+^ (Senn et al., 2001). Another report showed that the V336I and R591C mutants of HvHAK1 improved K^+^ nutrition and increased Na^+^ tolerance of yeast cells by increasing the *V*_max_ of K^+^ uptake (Mangano et al., 2008). In Arabidopsis, the L776H mutation of AtHAK5 increased the *V*_max_ of Rb^+^ uptake without affecting the *K*_m_ value, improving growth of yeast cells under low K^+^ (Rubio et al., 2000). In *Physcomitrella patens*, the R443S, AQQP/VQP, L603H and G606E mutations in PpHAK1 decreased the *K*_m_ and, with the exception of R443S, increased the *V*_max_ for Rb^+^ uptake in yeast. All these PpHAK1 mutants improved yeast growth at low K^+^ (Garciadeblas et al., 2007). All these studies either used site-directed (Senn et al., 2001) or random mutagenic PCR (Mangano et al., 2008) or took advantage of the occurrence of spontaneous mutations in DNA (Rubio et al., 2000; Garciadeblas et al., 2007). Here, to increase the chances of isolating new mutations with higher transport selectivity, a modified mutagenic PCR approach was successfully used. According to it, different unbalanced mixtures of dNTPs were used in different reactions to favor the occurrence of transitions or transversions. To increase the selective pressure, mutants were selected on the presence of high Na^+^ in the yeast strain 9.3, which is highly sensitive to Na^+^ because of deletion of the endogenous K^+^ uptake systems (TKR1, TRK2) as well the Na^+^ extruding ATPases (ENA1-ENA4) (Bañuelos et al., 1995). In addition, the L776H mutant instead of WT AtHAK5 was used as a starting material, to generate new mutants that increase Na^+^ tolerance beyond that of the L776H mutant.

Here we show a mutation, F130S, which importantly affects the kinetics of K^+^ uptake of a HAK5 transporter, leading to improved growth at low K^+^ as well as enhanced tolerance to Na^+^ and Cs^+^ (Table 1 and Figure 1). The F130S mutation in the AtHAK5 transporter increases in yeast cells 100-fold the affinity for Rb^+^ uptake, two-fold the *V*_max_ of Rb^+^ uptake (Table 2 and Figure 6) and 7-fold the maximal rate of K^+^ depletion (4.2 ± 0.5 vs 0.6 ± 0.3 nmol mg^−1^ min^−1^ for F103S and WT respectively). In addition, important reductions in the *K*_i_ values for K^+^, Na^+^, and Cs^+^ are observed (Table 2). The changes in the kinetic parameters promoted by the F130S mutation lead to yeast cells that were able to maintain higher concentrations of K^+^ and lower concentrations of Na^+^ at high external Na^+^ concentrations (Figure 2), as well as higher concentrations of K^+^ and lower of Cs^+^ in the presence of Cs^+^ (Figure 4). Overall, yeast cells expressing the mutant showed higher K^+^/Na^+^ (Figure 3) and K^+^/Cs^+^ (Figure 5) ratios, crucial parameters for salt and Cs^+^ tolerances in yeast as well as in plants (Gaxiola et al., 1992; Maathuis and Amtmann, 1999; Gisbert et al., 2000; Hampton et al., 2004). Importantly, the changes mediated by the F130S amino acid substitution occurred without increasing the amount of transporter protein in yeast membranes (Figure 7).

The results summarized above suggest that the F130 residue of the AtHAK5 transporter may affect a domain that is involved in K^+^ binding and/or transport from the external solution to the cytoplasm. K^+^, Na^+^, and Cs^+^ competitively inhibit HAK5-mediated Rb^+^ uptake and they are transported through HAK5 transporters (Santa-María et al., 1997; Rubio et al., 2000; Qi et al., 2008). Therefore, the domain involved in K^+^ binding of this type of transporters is also probably involved in Rb^+^, Na^+^, and Cs^+^ binding. Then, a mutation in the K^+^ binding site that increases the affinity for K^+^ is expected to produce a similar effect on the affinities for Rb^+^, Na^+^, and Cs^+^, which is the effect that can be deduced from the data presented here (Table 2). The idea that the F130S mutation is affecting a domain involved in the binding of K^+^, and therefore the binding of Na^+^ and Cs^+^, is further supported by the lower accumulation of Na^+^ (Figure 2B) and Cs^+^ (Figure 4B) observed in yeast expressing this mutant in comparison with those expressing WT AtHAK5.

**Figure 8:**
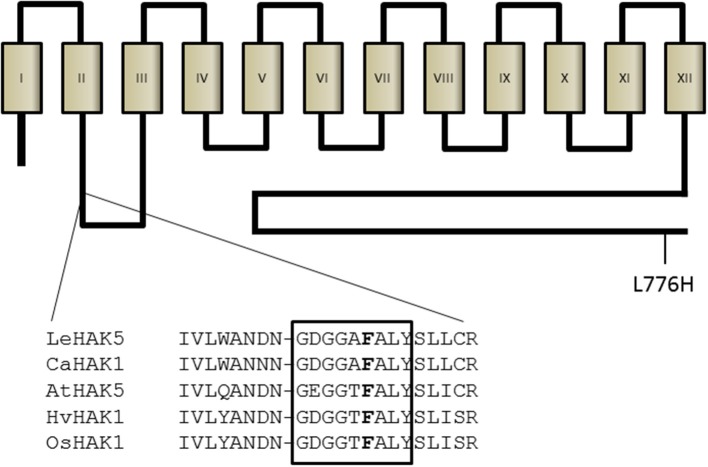
**Model of AtHAK5 topology and location of mutations**. The proposed model for AtHAK5 protein is shown with 12 transmembrane domains and a long cytoplasmic C-terminus. The F130 residue is located between the second and the third transmembrane domains, in a highly glycine-rich conserved region among HAK transporters. Shown is the alignment of this conserved region in representative high-affinity HAK K^+^ transporters of different species. The location of the L776H is also shown.

Recently, the topology of a bacterial member of the HAK family of transporters, the *Escherichia coli* Kup system, has been determined (Sato et al., 2014). According to this study, this type of transporters is composed of 12 transmembrane domains with the N- and C-termini facing the cytoplasmic side. The reported structure confirms the initial model proposed for AtHAK5 (Rubio et al., 2000), which was based on algorithmic prediction of transmembrane domains. This structure may be very likely extended to all members of the HAK family (Rodríguez-Navarro, 2000). Although all HAK transporters may have a similar structure they do not show extensive conserved regions. However, a study that included plant, fungal and bacterial representatives found 13 conserved glycine residues (Rodríguez-Navarro, 2000), which have been found later in many other HAK transporters. Three out of these 13 glycine residues are located in the GEGGT**F**ALY domain, highly conserved among HAK transporters and that contains the F130 residue proposed here as important for K^+^ affinity (Figure 8). Importantly, the selectivity pore of K^+^ channels contains the glycine-rich motif GYG (Heginbotham et al., 1992) and related motifs are found in the proposed pore regions of HKT-TRK transporters (Durell and Guy, 1999; Mäser et al., 2002). Interestingly enough, mutations that affect glycine residues in several K^+^ transport systems that belong to different families, greatly affect their capacity to mediate K^+^ transport. The paradigmatic example is the removal of the GYG motif of a K^+^ channel that modifies its selectivity (Heginbotham et al., 1992). Other examples are the G91S mutation in TaHKT2.1 [former TaHKT1 (Platten et al., 2006)], that abrogates K^+^ permeability (Mäser et al., 2002) or the G61S mutation in HvHAK1 that reduces the affinity and the *V*_max_ for K^+^ uptake (Senn et al., 2001). In conclusion, it is tempting to speculate that the GEGGT**F**ALY domain and other glycine-rich regions found along HAK proteins (Rodríguez-Navarro, 2000), are involved in shaping the pore region for K^+^ binding, a very attractive hypothesis that needs further investigation. Modeling studies together with ion current measurements in heterologous systems should help to further characterize the structure-function relationships of this type of transporters. It is worth noting that the F130 residue studied here is conserved among almost all members of the HAK family identified so far in plants (Nieves-Cordones, personal communication). Importantly, this residue is present in all HAK transporters homologous to the high-affinity HAK5-type transporters identified in different plant species whose sequence has been deposited in the Genebank (Figure S1).

Interestingly, HAK5-type transporters can also mediate low-affinity Na^+^ uptake (Santa-María et al., 1997) and high-affinity Cs^+^ uptake (Rubio et al., 2000) and therefore, they may be involved in the accumulation of toxic Na^+^ or Cs^+^. The contribution of HAK5-type transporters to low-affinity Na^+^ uptake and Na^+^ accumulation may be low for two main reasons. Firstly, salinity reduces the number of HAK5 transporters in the plasma membrane because high external Na^+^ reduces the expression of the encoding genes (Nieves-Cordones et al., 2008, 2010; Alemán et al., 2009). Secondly, although yet unidentified at the molecular level, it is proposed that the main pathways for low-affinity Na^+^ uptake are non-selective cation channels (Amtmann and Sanders, 1999; Tester and Davenport, 2003). Thus, although HAK5 transporters may be crucial to sustain K^+^ nutrition under K^+^-deficiency and salinity (Nieves-Cordones et al., 2010), they may not be relevant in Na^+^ accumulation.

However, HAK5 transporters may play an important role in the accumulation of toxic Cs^+^. Two main pathways have been proposed for Cs^+^ uptake in plants. One is a Ca^2+^-sensitive pathway that operates in K^+^-sufficient plants, and the other is insensitive to Ca^2+^ and it is only present in K^+^-deprived plants (Heredia et al., 2002; Hampton et al., 2004; Caballero et al., 2012). This Ca^2+^-insensitive pathway is very likely mediated by HAK5 transporters. Importantly, high Cs^+^ concentrations may lead to K^+^ starvation and induction of HAK5 genes, and HAK5 transporters may mediate Cs^+^ uptake even in K^+^-sufficient plants (Adams et al., 2013). Thus, furnishing plants with mutated versions of HAK5 that reduce the accumulation of Cs^+^ could contribute to tolerance to this toxic cation. Since Cs^+^ is a non-essential and rare cation in nature (White and Broadley, 2000), natural selection of HAK5 transporters with a high K^+^/Cs^+^ discrimination may have not been occurred. Thus, a biotechnological approach based on random mutagenesis and selection by function, should be a promising alternative.

The selection of HAK5 mutants in yeast, as shown here, results instrumental to identify functional domains of the transporter. However, a mutant may promote higher rates of K^+^ uptake in yeast but not in plants. This can occur for example if the mutation produces a codon which is preferred in yeast and that leads to an increased amount of the protein in the fungus (Sharp et al., 1986; Campbell and Gowri, 1990). In this sense, mutants that show an increase in the affinity (Table 2) without importantly increasing the *V*_max_ nor the amount of protein in yeast (Figure 7) as the F130S, may provide more promising tools for biotechnological purposes.

Mutated versions of HAK5-type transporters, as the one described here, may provide biotechnological tools to improve K^+^ nutrition of plants under K^+^ deficiency or under certain abiotic stress conditions (Niu et al., 1995). Mutated HAK5 transporters can be expressed in plants to increase K^+^ acquisition from low concentrations. In addition, the expression of these mutants can also provide a means of acquiring K^+^ under salinity or Cs^+^-polluted soils, conditions that are known to induced K^+^ deficiency (Botella et al., 1997; Carden et al., 2003; Hampton et al., 2004; Shabala et al., 2006) and to reduce HAK-mediated accumulation of toxic Cs^+^.

### Conflict of interest statement

The authors declare that the research was conducted in the absence of any commercial or financial relationships that could be construed as a potential conflict of interest.
