# Supplementary material for: The F130S point mutation in the Arabidopsis high-affinity K+ transporter AtHAK5 increases K+ over Na+ and Cs+ selectivity and confers Na+ and Cs+ tolerance to yeast under heterologous expression
Source: Front Plant Sci. 2014 Sep 2;5:430. doi: 10.3389/fpls.2014.00430 (PMC4151339; doi:10.3389/fpls.2014.00430)
Supplement: Supplementary file 1 [file Table1.DOCX]

**Table S1. Chemical composition of reactions for random mutagenic PCR**

| **Reactive** | **Transitions** | **Transversions** |
| --- | --- | --- |
| DNA | 15ng | 15ng |
| dCTC | 1 mM | 1 mM |
| dTTP | 1 mM | 1 mM |
| dATP | 0,2 mM | 1 mM |
| dGTP | 1 mM | 0,2 mM |
| Primers | 30 pmol | 30 pmol |
| MgCl2 | 7 mM | 7 mM |
| MnCl2 | 0.5 mM | 0.5 mM |
| KCl | 50 mM | 50 mM |
| Final Volume | 50 µL | 50 µL |
