# Supplementary material for: The F130S point mutation in the Arabidopsis high-affinity K+ transporter AtHAK5 increases K+ over Na+ and Cs+ selectivity and confers Na+ and Cs+ tolerance to yeast under heterologous expression
Source: Front Plant Sci. 2014 Sep 2;5:430. doi: 10.3389/fpls.2014.00430 (PMC4151339; doi:10.3389/fpls.2014.00430)
Supplement: Supplementary file 2 [file Presentation1.PDF]

```
TaHAK1          DG---IKDRDDLLGVLSLILYTLIIIPMLKYVFIVLYANDNGDGGTFALYSLISRYAKIR 130
AetHAK1         DG---IKDRDDLLGVLSLILYTLIIIPMLKYVFIVLYANDNGDGGTFALYSLISRYAKIR 130
HvHAK1          DG---IRNRDDLLGVLSLILYTLIIIPMLKYVFIVLYANDNGDGGTFALYSLISRYAKIR 87
ZmHAK1          DG---IKYNDDLLGVLSLIIYTLIIIPMLKYVFVVLYANDNGDGGTFALYSLISRYAKIR 137
AlHAK1          NG---IKNNDDLLGVLSLIIYTLIIIPMLKYVFIVLYANDNGDGGTFALYSLISRYAKIR 132
OsHAK1          DG---IGHRDDLVGVLSLILYTLIIIPMLKYVFIVLYANDNGDGGTFALYSLISRYAKIR 140
AetHAK5         GG---IKDTDDLLGVMSLIIYTVLLLPLMKYCFIVLRANDNGDGGTFALYSLISRYARIS 148
TuHAK5          GG---IKDTDDLLGVMSLIIYTVLLLPLMKYCFIVLRANDNGDGGTFALYSLISRYARIS 149
OsHAK5          NG---IKDTNDILGVMSLIIYTVVLLPLIKYCFIVLRANDNGDGGTFALYSLISRYARIS 146
ZmHAK5          NG---INNTDDLLGVMSLIIYTVILLPLIKYCFIVLRANDNGDGGTFALYSLISRYARVS 143
CaHAK1          DK---IGHKDDILGVLSLIIYTIILVPMTKYVFIVLWANNNGDGGAFALYSLLCRYAKVS 144
SlHAK5          DE---IKHKDDILGVLSLIIYTIMLVPMTKYVFIVLWANDNGDGGAFALYSLLCRYAKVS 141
AtHAK5          DG---INDKDDVVGVLSLIIYTITLVALLKYVFIVLQANDNGEGGTFALYSLICRYAKMG 143
ThHAK5          EG---INDKDDVIGVLSLIIYTLTLVALLKYVFIVLQANDNGEGGTFALYSLICRYAKTG 148
PpHAK1          SG---IKTNDDILGVLCLIIYTIIATPLVKYIFIVLRANDNGEGGTFALYSLICRHVKLS 174
VfHAK1          KVP--INSDNDVLGALSLVMYTIALIPLAKYVFIVLKANDNGEGGTFALYSLICRYANVN 178
CeaHAK1         EDIHHSDTNEEIYGVLSFVFWTLTLIPLLKYVFIVLRADDNGEGGTFALYSLLCRHARVS 106
McHAK1          EDIQHSESNEEIYGVLSFVFWTLTLIPLLKYVFIVLRADDNGEGGTFALYSLLCRHARVS 106
NtHAK1          EDIQHSESDDEIFGVLSFVFWTLTLIPLLKYVFIVLRADDNGEGGTFALYSLLCRHARVS 116
NrHAK1          EDIQHSESNDEIFGVLSFVFWTLTLIPLLKYVFIVLRADDNGEGGTFALYSLLCRHARVS 116
AaHAK1          EDILHSETNEEIFGALSFIFWTLTLVPLLKYVFIVLTGDDNGEGGTFALYSLLCRHARVS 108
SeHAK1          EDIEHSDTSEEIYGVLSFVFWTLTIIPLLKYVFIVLRADDNGEGGTFALYSLLCRHARVN 109
CnHAK1          EDIEHSETNEEIFGVLSFIFWTLTLVPLVKYVFIVLRADDNGEGGTFALYSLICRHARVG 109
                         ::: *.:.::::*:   .: ** *:** .::**:**:******:.*:..
```

Figure S1. Alignment of the conserved region containing the AtHAK5 F130 residue of all HAK5-type transporters identified in the Genebank. Boxed is the highly conserved domain among HAK transporters which is rich in glycine residues and contains the F130 residue.
